# Comprehensive View of Magnesium Physiology

**DOI:** 10.2174/0118715303364703250224053714

**Published:** 2025-04-21

**Authors:** Shagufta Jawaid, Samya Shams, Damini Kharb, Omar A. Al-Bar, Mustafa Zeyadi, Ibrahim Sultan AL Hazmi, Vikas Kumar, Firoz Anwar

**Affiliations:** 1 Department of Pharmacy Practice, School of Pharmaceutical Sciences, Shri Guru Ram Rai University, Dehradun, Uttarakhand, India;; 2 Department of Biochemistry, Faculty of Science, King Abdulaziz University, Jeddah, 21589, Saudi Arabia;; 3 Natural Product Drug Discovery Laboratory, Department of Pharmaceutical Sciences, Faculty of Health Sciences, Sam Higginbottom University of Agriculture, Technology & Sciences, Prayagraj, Uttar Pradesh, 211007, India

**Keywords:** Magnesium, physiology, hypomagnesaemia, transporters, human diseases, Mg disorders

## Abstract

Significant progress has been observed in recent decades, elucidating the biochemical, cellular, and molecular pathways that regulate the 10% inorganic ions, such as Na, K, Ca, HCO_3_, Cl, and fluid balance in both health and diseases. However, magnesium (Mg), a forgotten electrolyte, despite its critical role as the calcium antagonist found in nature with clinically relevant data on Mg disorders, was one of the least used ions till the late twentieth century.

The underappreciation of the clinical importance of Mg may stem from a lack of detailed knowledge about its regulation at the subcellular and systemic levels. Recent technologies and scientific advancements in identifying Mg-specific ion channels and transporters, alongside a more profound understanding of biochemical, physiological, and hormonal mechanisms regulating Mg homeostasis, have opened new ways and renewed interest in magnesium research in human science, shedding light on its critical multidimensional role in various biological processes and offering new insight into its therapeutic potential.

This comprehensive review delves into the latest advancements regarding the physiological functions of Mg, emphasising hypomagnesaemia as the most clinical manifestation of Mg imbalance. It covers key aspects, such as the physiology of Mg transporters, the renal mechanism in Mg homeostasis, and integrated regulatory systems governing its balance. Additionally, it addresses Mg deficiency, highlighting the intricate interplay between Mg, Ca, and K deficiencies, drug-induced Mg depletion, and non-pharmacological aetiologies. The review also looks at how common magnesium deficiencies are, how to diagnose them, and what they mean for patients. It also looks at how magnesium supplements can help with a number of medical conditions, giving a full picture of the important biological role of magnesium.

## 1 INTRODUCTION

Magnesium is an important inorganic ion that naturally occurs as an Mg^2+^ ion in the human body [1]. It is the fourth most abundant ion in human body and exists as a vital electrolyte for sustaining cellular and organ utility [2]. It is found in all living things, from plants to highly developed animals. It is necessary for fitness and long life because it is involved in over 300 enzyme reactions [3], some of which are linked to making energy, DNA repair [4, 5], and protein synthesis [6]. It is also a necessary cofactor for ATP [6], which is the building block of energy in cells. Penetrating through the blood-brain barrier, it also plays a significant role in neurological development, as well as the working of the brain and spinal cord network [7].

Magnesium is an important mineral for many cellular and physiological processes, mostly because it binds to nucleotides and controls how enzymes work [8]. ATPase responses necessitate Mg^2+^-ATP, counting those involved in RNA and DNA utilities [9]. Magnesium is involved in the mechanism of neuromuscular function. It supports the release of neurotransmitters and plays a role in the function of ion channels involved in nerve signal transmission [10], helps preserve the reliability of the cerebrovascular barrier, and inhibits oxidative stress-related swelling. Magnesium also regulates cardiac rhythm [11], sustains myelin sheaths and myelinated axons [12], modulates vascular tone [13], influences hormone secretion [14], and regulates N-methyl-D-aspartic acid (NMDA) release within the central nervous system [15]. Additionally, magnesium improves excitotoxic damage *via* obstructing N-methyl-D-aspartic acid sensor action along with extreme calcium (Ca) invasion [16].

**Fig. (1):**
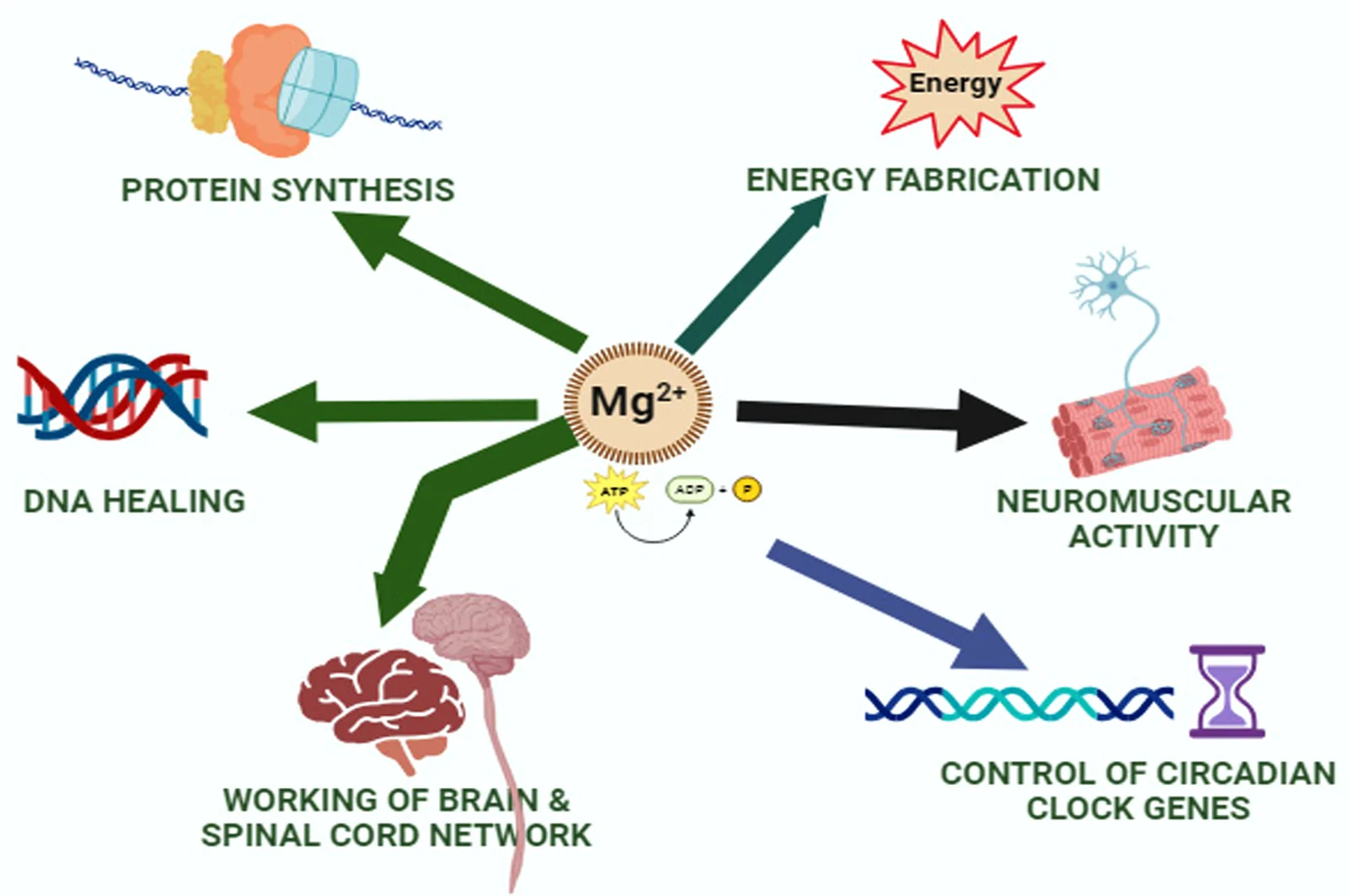
Essential Roles of Magnesium (Mg^2+^) in Biological Processes: Magnesium (Mg^2+^) is crucial for numerous cellular functions, including protein synthesis, DNA repair, and energy production through ATP generation, supporting neuromuscular activity by stabilising nerve and muscle function, influencing brain and spinal cord networks, and regulating circadian rhythms by controlling clock genes. A lack of magnesium can mess up these processes, affecting metabolic energy, the functioning of the nervous system, and the repair of cells. This shows how important magnesium is for overall health.

Magnesium is a subsequentmessenger intricately involved in intracellular signaling and servesas a controller of circadian clock genes [17], which control the circadian rhythm in biological systems (Fig. **1**).

## 2 PHYSIOLOGY OF MAGNESIUM CARRIERS

Important magnesium pathways in the direction of biological unit function and stimulus levels of magnesium inside the cell should be tightly controlled [18]. In the late 1950s, magnesium-specific carriers were first found in bacteria, fungi, and yeast [19]. They were named Mg transporters, and they keep magnesium levels in humans stable [20]. Their carriers were the primary magnesium transporters from ion channel subfamily M members 6 and 7 (TRPM6 and TRPM7) [10]. TRPM6 is expressed chiefly in the colon and distal intricate tubule of the kidney and is accountable for the uptake of magnesium inside the intestine and kidney (Table **1**) [21]. Scientists first understood what TRPM6 meant when they found that changes in this gene were linked to magnesium deficiency, secondary calcium deficiency (HSH), and other syndromes linked to magnesium deficiency. In mice, the homozygous deletion of Trpm6 is fatal to the embryo, while its heterozygous deletion predisposes to magnesium deficiency, which is refractory to magnesium supplementation [22]. Unlike TRPM6, TRPM7 is universally expressed and is vital for cell viability and life itself [23]. Homozygous Trpm7-knockout mice do not survive the embryonic stage, whereas Trmp7 heterozygotes have magnesium deficiency, diminished development, and vascular dysfunction [19]. Frequent features that affect TRPM6 and TRPM7 action have been defined as magnesiotropic (concerning magnesium regulation), comprising cutaneous/skin development [24], fibrocytedevelopment [25], uromodulin [26], adenosine diphosphate (ADP) ribosylation factor-like protein 15 [27], aldosterone [28], angiotensin II [29], bradykinin, and insulin [30]. Additional magnesium transporters include solute carrier family 41 members 1, 2, and 3 [31], as well as the magnesium-selective mitochondrial RNA splicing protein 2 (MRS2) [3]. MAGT1, initially defined as a magnesium transporter, is an organiser of N-linked protein glycosylation [32] that ultimately influences magnesium transport and homeostasis.

## 3 RENAL REGULATION OF MAGNESIUM HOMEOSTASIS

The process of magnesium (Mg) transfer in the kidney highlights different locations, transport mechanisms, and regulatory factors that either enhance or reduce magnesium reabsorption. Around 15% to 20% of Mg is taken back up passively through paracellular transport [33], which is pushed by the electrochemical gradient [34]. Volume reduction amplifies Mg reabsorption, while volume enlargement diminishes it [35]. Most of the magnesium reabsorption (60–70%) occurs in the Thick Ascending Limb (TAL) [36], which is passive and happens through tight junctions between cells. This process is regulated by the calcium-sensing receptor (CaSR) [37]. Hormones like calcitonin, glucagon, AVP (arginine vasopressin), and PTH (parathyroid hormone) make Mg reabsorption stronger [38]. On the other hand, diuretics like furosemide [39] and high plasma Mg or Ca concentrations weaken it. The Distal Convoluted Tubule (DCT) actively re-absorbs about 10% of Mg through TRPM 6 channels [40, 41] on the apical membrane and the Na/Mg exchanger on the basolateral membrane [42]. This process is controlled by hormones like PTH, oestrogen, insulin, and EGF (epidermal growth factor) [43]. PTH, calcitonin, vitamin D, and low plasma Mg levels enhance reabsorption, whereas high Mg or Ca levels and metabolic acidosis reduce it [44]. The collecting duct has minimal involvement in Mg reabsorption, with very limited transport occurring in this segment (Table **1**).

## 4 INTEGRATED MANAGEMENT OF MG HOMEOSTASIS

The body comprises of magnesium, with a bulk deposited within the osseous plus non-osseous matter [45]. The stability of magnesiumin the body is crucial to retaining the correct levels of magnesium, which is essential for several physiological functions [46]. Magnesium plays a significant role in muscle function, nerve transmission, bone health, and energy production [47]. Magnesium is the second most important ion in cells, after potassium [48]. In cells, more than 90% of magnesium is liganded [49]. Free magnesium occurs in simply 1 to 5% of cellular magnesium [50]. The intracellular concentration of free magnesium is 1.2 to 2.9 milligrams/decilitre, which is analogous to the extracellular concentration [51]. About 30% of the flowing magnesium in plasma is associated with proteins, typically through circulating fatty acids [52]. The corresponding mechanism of magnesium stability includes an intricate interplay between several organs and systems to sustain ideal magnesium levels in the body [53]. The body controls magnesium stability through a combination of dietary ingestion, absorption, and excretion [54]. Consequently, patients with chronically high levels of free fatty acids mostly have a lower blood magnesium concentration, and plasma magnesium levels are contrariwise relative to the threat of cardiovascular and metabolic diseases [55]. Changes in free fatty acids [56], EGFR [57], insulin, and aldosterone [13] levels may make magnesium levels in the blood less stable. Magnesium is regulated chiefly through three organs at the system level: the intestine, which consumes a stable diet rich in magnesium [58, 59], where dietary magnesium absorption is regulated and employed for several bodily functions; the bone, which stores magnesium as hydroxyapatite [60] and contributes to bone health through bone composition, bone mineralisation, calcium regulation, and bone remodelling [61]; and the renal organ, which controls magnesium elimination through filtration and reabsorption [62]. Electrolyte balance and suitable magnesium ingestion also inhibit kidney stones. They combine to create an intestine–osseous–renal alignment, which is accountable for the utilisation, alteration, and elimination of magnesium individually [63]. This imbalance raises pathophysiological concerns.

## 5 MAGNESIUM DEFICIENCY

**Fig. (2):**
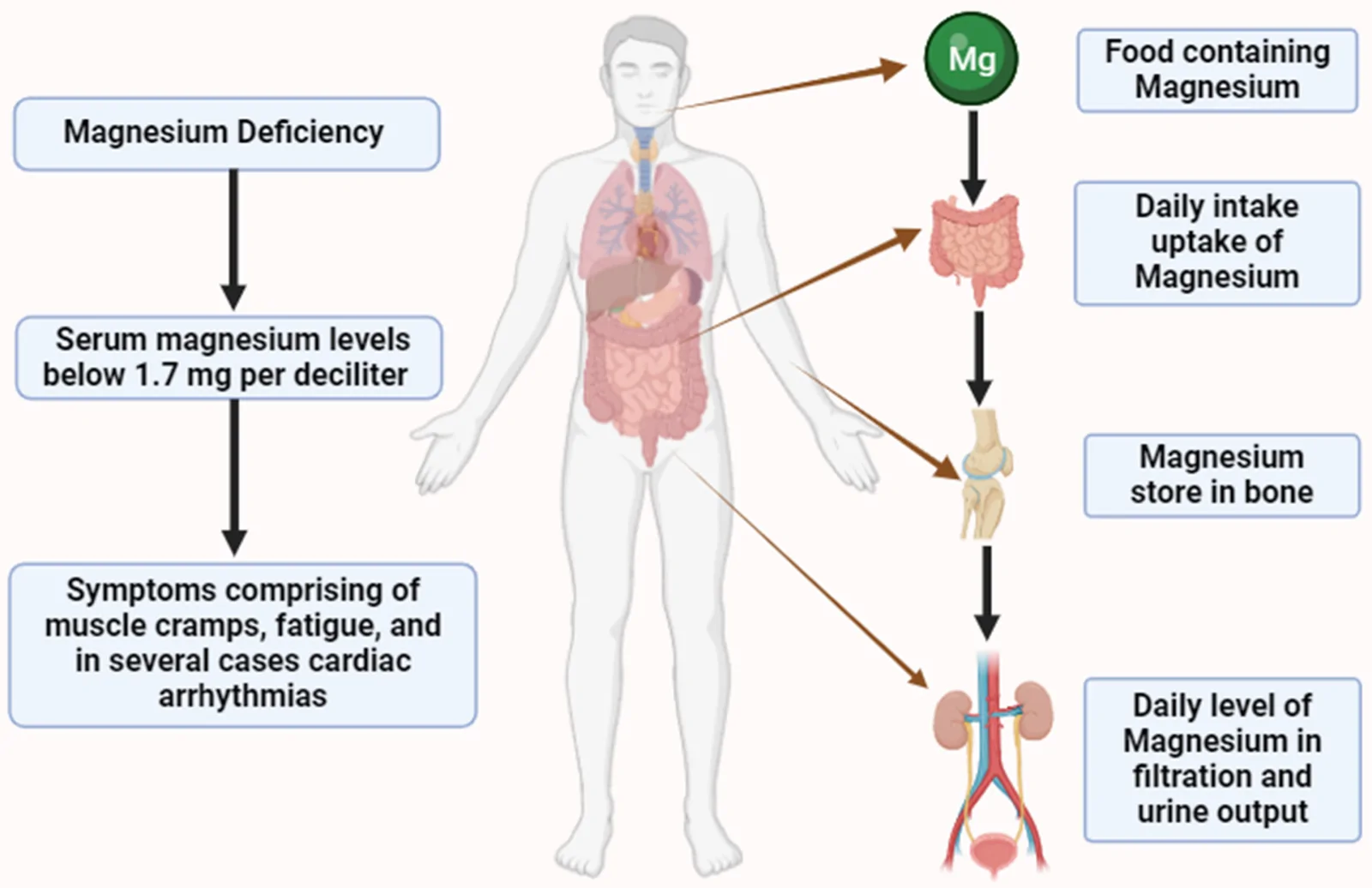
Mechanism of magnesium deficiency and its physiological ipacts: magnesium (Mg^2+^) from dietary intake is absorbed in the intestine and stored primarily in bones. The kidneys regulate Mg levels through filtration and urinary excretion. When serum Mg^2+^ levels fall below 1.7 mg/dL, deficiency symptoms manifest, including muscle cramps, fatigue, and, in severe cases, cardiac arrhythmias. Mg^2+^ deficiency results from inadequate intake, absorption issues, or excessive excretion, highlighting the importance of maintaining balanced Mg^2+^ levels for overall health.

Uncharacteristically low levels of magnesium in the blood might be due to gastrointestinal loss, renal loss, nutritional deficiency, prolonged alcoholism, and some medications [64]. The typical serum magnesium concentration in adults is 1.7 to 2.4 milligrams/decilitre [64]. Magnesium deficiency is defined as serum magnesium levels below 1.7 mg per decilitre [65]. Its efficiency can lead to several symptoms comprising clear cramps, fatigue, and, in several cases, cardiac arrhythmias (Fig. **2**) [66]. However, the majority of patients with marginal magnesium deficiency are asymptomatic [67]. It has been suggested that the low cutoff point for magnesium deficiency be raised because people have a chronic underlying magnesium deficiency when their serum magnesium levels are above 1.5 mg per decilitre (0.6 mmol per litre) [68]. However, this level remains controversial and requires further clinical validation. Magnesium deficiency exists in 15% of the general population, but its prevalence is amplified amongst type 2 diabetes patients [69] (10–30%) in addition to hospitalised patients (10–60%), particularly individuals in the ICU (>65%) [70]. Numerous cohorts provide evidence that magnesium deficiency increases the risk of death from all causes and cardiovascular causes [71].

So, a magnesium deficiency might go unnoticed because the magnesium level in plasma is not usually checked in the worst medical situations [72]. There are three levels of magnesium deficiency: mild (plasma magnesium level ranging from 1.2 to 1.6 mg/decilitre (0.50 to 0.69 mmol/L)), moderate (plasma magnesium level 0.9 to 1.1 milligrammes per decilitre (0.38 to 0.49 millimoles per litre), and severe (serum magnesium level <0.9 mg/dL (<0.38 mmol/L) [73]. In severe cases, symptoms may include neuromuscular irritability (spasms, seizures, and tremors), cardiovascular problems (arrhythmias and vasoconstriction], and metabolic disorders (insulin resistance and chondrocalcinosis) [19].

### 5.1 Interplay of Calcium, Potassium, and Magnesium Deficiency

Calcium deficiency is a condition that occurs when total serum calcium levels are lower than 8.5 milligrams per decilitre (2.12 millimoles per litre) [74] or free calcium levels are lower than 4.6 milligrams per decilitre (1.16 millimoles per litre) [75], as characterised in a previous study [76]. Magnesium deficiency often coexists with calcium deficiency. A magnesium shortage suppresses the release of PTH and declines renal sensitivity to it [77]. When the level of PTH drops, the kidneys cannot reabsorb as much calcium, which can cause calciuria and a secondary calcium deficiency [78]. Correction cannot resolve the calcium deficiency resulting from magnesium deficiency-induced hypoparathyroidism until the magnesium concentration returns to normal [79]. Potassium deficiency is a condition identified *via* lesser levels of potassium in the plasma (below 3.5 mEq/L) [80]. Symptoms of potassium deficiency comprise muscle weakness, respiratory dysfunction, cardiac arrhythmias, and rhabdomyolysis [81]. Potassium deficiency is common in patients with magnesium deficiency [82]. Magnesium reduction often leads to refractory potassium repletion, which only adjusts once the magnesium shortage is under control [13]. A lack of magnesium makes the kidneys lose more potassium by making the collecting duct release more potassium [42]. Lower magnesium levels inside cells stop the Na^+^/K^+^–ATPase pump from working and make more ROMK channels open, which causes the kidneys to lose potassium [83]. When magnesium and potassium interact, they also stimulate the Na^+^Cl^+^ co-transporter (NCC), which helps the body take back sodium [84]. A lack of magnesium lowers the amount of NCC by blocking an E3 ubiquitin-protein ligase known as neuronal precursor cell developmentally down-regulated 4-2 (NEDD4-2). A lack of potassium also stops NCC from activating [85]. When magnesium levels are low, continuous NCC down-regulation improves the distribution of Na^+^ at the distal end. This leads to kaliuresis and potassium deficiencies.

### 5.2 Drug-Induced Magnesium Deficiency

Several medication categories, such as microbicides, urine stimulants, biological substances, immune suppressants, acid suppressors, and malignancy treatments, might originate from magnesium degeneration and magnesium deficiency [86]. Long-term use of acid suppressants can lead to magnesium deficiencies in about 20% of patients since these effects depend on dosage [87]. PPIs lower the amount of magnesium that the gut takes in and are linked to changes in the gut microbiome and luminal pH [88]. Some patients may be more susceptible to PPI-related magnesium deficiency due to factors, such as the dosage and duration of PPI therapy [89], dietary magnesium intake, co-treatment with additional magnesium-losing medication, and changes in the gut microbiome [90]. Researchers describe how oral inulin amplifies gastrointestinal absorption to improve plasma magnesium levels in individuals with PPI-related magnesium deficiency [91]. Renal magnesium wasting accounts for the majority of medication-related magnesium deficiency cases [92]. Calcineurin inhibitors [93], cisplatin [94], EGF receptor (EGFR) antagonists (*e.g.*, cetuximab and erlotinib) [82, 95], and mammalian target of rapamycin inhibitors cause magnesium deficiency in 20 to 40% of patients receiving them, mainly through reduced TRPM6 and TRPM7 activity in the distal convoluted tubule (Table **2**) [96-104].

### 5.3 Non-pharmacological Aetiology of Magnesium Deficiency

Magnesium deficiency is the most common electrolyte irregularity related to prolonged alcohol use conditions [105]. Fundamental mechanisms comprise reduced magnesium consumption in undernourished individuals, amplified digestive damage, and magnesuria owing to alcohol-caused nephritic tubular impairment [106]. Individuals with alcohol use conditions frequently experience magnesium deficiency, which is often associated with liver dysfunction and a worse prognosis for their liver disease [107]. Individuals with type 2 diabetes mellitus typically exhibit magnesium deficiency [108]. Urological magnesium degenerative conditions have led to an increase in serum globulin levels in the haematological fluid, which could potentially be the cause of magnesium deficiency [109]. Insulin stimulates TRPM6 action in the distal convoluted tubule. Accordingly, insulin resistance leads to diminished renal magnesium reabsorption and increased magnesuria [110]. Chronic stress can also lead to depleted magnesium levels [111]. Abnormal magnesium homeostasis is also linked to cardiovascular disease. In the heart, magnesium deficiency causes susceptibility to electrical irritability and arrhythmias, counting atrial fibrillation, torsade de pointes, and long QT syndrome. In the haemodynamic network, reduced magnesium levels are related to endothelial impairment, haemodynamic compression, and amplified haemodynamic tone, and fibrosis, typical traits of elevated blood pressure [112]. These vascular properties include modifications in TRPM7 movement and diminished magnesium influx in vascular cells and are particularly manifested in hyperaldosteronism [113] (Table **3**).

### 5.4 Inherited Magnesium Deficiency

Genetic magnesium deficiencies are caused by an inability to absorb or retain magnesium due to a genetic mutation [114]. It has been found that pathogenic variations in genes that code for magnesium transport pathways and their controllers are thought to be the cause of familial magnesium deficiency in almost 80% of patients [115]. Maximum genetically recognised bases for magnesium deficiency are due to magnesium reabsorption in the convoluted tubule [116]. Alterations in TRPM6 and TRPM7 subunits are principal to HSH [117]. Hypoparathyroidism, which triggers reduced intracellular magnesium levels in the parathyroid gland and damages PTH secretion, describes calcium deficiency in such patients [118]. Due to TRPM6 activity, changes in EGF and EGFR that are harmful, can lead to magnesium deficiency and kidney magnesium degeneration [119]. Pathogenic variations in CNNM2 result in magnesium deficiency, seizures, and cognitive deficiency [120]. The distal convoluted tubule expresses CNNM2, which regulates basolateral magnesium extrusion, although the molecular mechanism remains indistinguishable [36]. Epidermal nerve syndrome is a rare form of magnesium deficiency that is caused by changes in the SLC12A312A3 gene. This gene codes for a protein that helps the kidneys move sodium and chloride [121]. However, patients with Gitelman’s syndrome exhibit magnesium deficiency, potassium deficiency, and metabolic alkalosis [122]. It is hard to explain why this syndrome has a magnesium deficiency, but preclinical data show that atrophy of the distal convoluted tubule, which happens when NCC is not working right, may lead to less magnesium being reabsorbed. People who have nephrocalcinosis because of changes in the CLDN16 and CLDN19 genes often have low magnesium levels, a lot of calcium in their urine, and calcium deposits in their kidneys [123].

## 6 MAGNESIUM DEFICIENCY: PREVALENCE AND CLINICAL EVALUATION

Scientific significance is emphasised through the outcomes that decrease plasma magnesium grade, which are related to elevated occurrences of casualties and hospital stays, especially potassium deficiency [124]. Of scientific application, plasma magnesium is not a consistent indicator of the entire content in tissues, contributing to less than 1% only in plasma [125]. Organised diminution–satiation works of literature in metabolic components revealed that although plasma magnesium absorption might be regular, intracellular stocks can be exhausted [126]. So, using blood magnesium levels without taking into account magnesium intake from food and loss through the kidneys might not fully reflect magnesium deficiency in a clinical setting [127]. Assessment of magnesium inadequacy in the medical background involves a systemic approach that includes evaluating clinical signs and symptoms, conducting relevant laboratory tests, and identifying underlying causes [128]. The amount of overall plasma magnesium is the regular method to discover the magnesium position in the medical background [129]. It offers a rapid assessment of short-term fluctuations in magnesium status but may under-evaluate the whole-body content of magnesium [130]. Endogenous features (hypoalbuminemia) and exogenous features (hemolyzed samplings and sample tube anticoagulants (*e.g.*, EDTA) can impact magnesium quantities and the necessity of measuring them when blood results are inferred. Serum-ionised magnesium can also be measured, but its clinical effectiveness is unclear [131]. When magnesium deficiency is analysed, the reason is generally specious from the patient’s history [132]. However, in the absence of a clear fundamental cause, it is crucial to distinguish between renal and gastrointestinal magnesium losses using specific diagnostic techniques like 24-hour magnesium elimination, fractional magnesium elimination, and the magnesium-loading test.

## 7 MAGNESIUM: CHEMICAL PROPERTIES, BIOLOGICAL ROLES, AND REGULATORY MECHANISMS

Mg is an essential mineral with critical biochemical roles in the human body [5]. It is characterised by unique chemical properties that facilitate its widespread use in physiological functions [48]. As a divalent cation (Mg^2+^), magnesium has a small ionic radius and a high charge density [133]. This makes it easy for it to interact with biomolecules like enzymes, nucleic acids, and proteins [134]. This substance is an important part of many enzyme systems, especially those that make proteins, DNA, and RNA [96]. Its role in these systems shows how important it is to cells. Magnesium is essential for maintaining the structural integrity of cells and tissues. It stabilises the structure of ATP (adenosine triphosphate) [135]—a crucial molecule for energy transfer in cells—by binding to its phosphate groups. Mg^2+^ also changes the electrical activity across cell membranes, especially in cells that are easily excited, like neurones and muscle cells [136]. It does this by controlling ion channels, like calcium and potassium channels [137]. This regulation of ion flow is critical for maintaining membrane potential and normal neuromuscular function. Approximately 60% of magnesium in the body is stored in the bones [138], contributing to bone structure alongside calcium and phosphorus. Another 20% is found in muscles [139], while the remainder resides in soft tissues and bodily fluids, including the blood. Even though magnesium is found in large amounts, only 1% of the body's magnesium is in the bloodstream [140]. This shows that tissue storage and serum levels are tightly controlled. Magnesium homeostasis is maintained through various transport proteins and ion channels that control its movement across cell membranes. Transient Receptor Potential Melastatin (TRPM6 and TRPM7) are two important transporters that help Mg^+^ get into epithelial and other cells [10]. Efflux, the process of magnesium exiting cells, is primarily mediated by Na^+^/Mg^2+^ exchangers [141]. Hormones like insulin and parathyroid hormone (PTH) also help keep magnesium levels stable [142] by making it easier for cells to take in magnesium or increasing its reabsorption in the kidneys. Dietary magnesium is primarily obtained from leafy greens, nuts, seeds, and whole grains [143]. The body absorbs about 30–50% of ingested magnesium [21], primarily in the small intestine. This is regulated by the body’s current magnesium status and the availability of TRPM6 channels [144]. Excess magnesium is excreted through the kidneys, which finely tune reabsorption to maintain magnesium balance. When magnesium levels are high, urinary excretion increases to avoid toxicity. Magnesium imbalances can lead to various pathophysiological conditions. Hypomagnesaemia or low serum magnesium, is usually caused by not getting enough magnesium, having trouble absorbing it, or losing too much magnesium through the kidneys. It can cause muscle weakness, heart rhythm problems, and neurological problems [145]. On the other hand, hypermagnesemia (high serum magnesium levels) is less common and usually happens in people with kidney disease [19] because their kidneys cannot get rid of excessive magnesium properly. Symptoms of hypermagnesemia include lethargy, hypotension, muscle weakness, and, in severe cases, respiratory depression and cardiac arrest [146]. Mg chemical characteristics enable it to support many physiological functions, from energy metabolism to muscle and nerve function. Proper regulation of magnesium consumption, absorption, cellular transport, and excretion ensures its balance within the body, underscoring its importance for health and well-being. Problematic changes in magnesium levels, especially hypermagnesemia, show how important it is for the body to keep magnesium levels in check.

## 8 THERAPEUTIC ROLE OF MG

Even though there are numerous conditions in which magnesium has been involved, there are a few medical illnesses in which magnesium is the therapeutic agent of choice [147]. Clinical vignettes include cases of torsades de pointes, acute asthma exacerbations, and preeclampsia or eclampsia. Magnesium treatment is recommended for patients with torsades de pointes who do not respond to beta-blockers [19].

Intravenous magnesium sulphate is recommended by clinical guidelines for people with severe asthma flare-ups that do not get better with standard treatment and for people whose asthma is life-threatening [148]. When nebulised magnesium sulphate is mixed with inhaled β2-agonists and ipratropium bromide, it may improve lung function even more and lower the number of hospital stays [149]. The beneficial effects of magnesium in asthma likely stem from calcium channel blockade in the bronchial smooth muscle, which subsequently leads to bronchodilation [150]. International clinical guidelines on the treatment of hypertension in pregnancy prescribe magnesium sulphate to inhibit seizures in women diagnosed with eclampsia [151]. Women diagnosed with preeclampsia who have proteinuria and serious hypertension or hypertension with neurologic indications must be treated with magnesium sulphate to prevent eclampsia [152]. It is possible for magnesium sulphate to help with these conditions, but it is not clear exactly how magnesium works to help patients [153].

## CONCLUSION

Magnesium is an essential electrolyte that is frequently suboptimally considered in clinical medicine [154]. It is often not evaluated as part of routine electrolyte screening. Magnesium deficiency is often asymptomatic [155]. While the exact mechanisms regulating magnesium levels in the body are still not fully understood, there have been advancements in understanding how magnesium is controlled in the kidneys [156].

This awareness is associated chiefly with the gene screening panels and whole-exome sequencing, which have recognised new genes that initiate rare forms of genetic magnesium deficiency [157]. Various drugs cause magnesium deficiency. Magnesium deficiency is typical in admitted patients and poses a risk for an extended ICU stay [158]. Magnesium deficiency should be addressed with magnesium supplement therapy in the form of organic salt preparation [159]. Although much remains to be understood about magnesium and its role in health and disease, the field has progressed, and clinicians should be more attuned to the significance of magnesium in clinical medicine [160].

## Figures and Tables

**Table 1:** The table categorizes kidney regions by Mg reabsorption location, mechanism, and regulation. Conditions that increase reabsorption, such as parathyroid hormone (PTH) and low plasma Mg, and those that reduce it, like diuretics or high plasma Mg. Key locations are involved in Mg reabsorption include the proximal tubule, thick ascending limb, distal convoluted tubule, and collecting duct.

**Location in Kidney**	**Transport Mechanism**	**Details**	**Regulation**	**Amplify Reabsorption**	**Diminish Reabsorption**
**Proximal Tubule**	Paracellular (passive)	15-20% of filtered Mg is reabsorbed passively between cells, determined by the electrochemical gradient.	-	Volume reduction	Volume enlargement
**Thick Ascending limb (TAL)**	Paracellular (passive)	60-70% of Mg reabsorption take place here through tight junctions, determined by lumen positive voltage.	Controlled by calcium-sensing receptor (CaSR).	PTH, calcitonin, glucagon, AVP, Reduce plasma (Mg), Metabolic alkalosis	Furosemide and related loop diuretics, Mannitol, High plasma (Mg^2+^) or (Ca^2+^)
**Distal Convoluted Tubule (DCT)**	Transcellular (active)	10% of Mg is reabsorbed actively *via* TRPM 6 channel on apical membrane and basolateral Na/Mg exchanger.	Controlled by PTH, estrogen, insulin and EGF.	PTH, calcitonin, glucagon, estrogens, vitamin D, Metabolic alkalosis, Low plasma (Mg^2+^), Amiloride	High plasma (Mg^2+^) or (Ca^2+^), Metabolic acidosis, K^+^ or phosphate depletion
**Collecting Duct**	Minimal reabsorption	Very limited Mg transport in this segment.	-	-	-

**Table 2:** Drug-Induced Magnesium Deficiency: The table summarizes drug classes that cause Magnesium (Mg) depletion *via* mechanisms like increased urinary excretion, reduced absorption, or renal reabsorption impairment. Clinical considerations emphasize Mg monitoring, particularly for patients with chronic conditions or on prolonged therapies. Diuretics and proton pump inhibitors commonly reduce Mg in long-term use, while drugs like antineoplastics and calcineurin inhibitors require vigilant renal and Mg level monitoring to manage deficiency risks. References detail each drug’s specific efforts.

**Drug Class**	**Examples**	** Mechanism of Mg Depletion **	**Clinical Consideration**	**References**
Diuretics	Thiazides, Furosemide	Increase urinary excretion of Mg	Common in prolonged use	[97]
Proton Pump Inhibitors	Omeprazole, Esomeprazole	Reduced intestinal absorption of Mg	Observed in chronic use	[89]
Aminoglycosides	Gentamicin, Tobramycin	Nephrotoxicity leading to renal Mg wasting	Through monitoring of RFT and Mg level during therapy	[98]
Antineoplastics	Cisplatin	Nephrotoxicity renal Mg loss	Frequent monitoring of electrolytes and KFT	[99]
Calcineurin Inhibitors	Cyclosporine, Tacrolimus	Impaired renal Mg reabsorption	Relevant in transplant patients	[100]
Beta-2-Agonists	Albuterol, Salbutamol	Shift Mg Intracellularly, lowering serum levels	Exacerbate Arrhythmias	[101]
Antifungal	Amphotericin B	Renal tubular damage- Mg loss	Frequent monitoring of Mg and other electrolytes	[102]
Antiviral Drugs	Adefovir, Tenofovir	Nephrotoxicity leading to Mg wasting	Important in HIV or HBV patients on chronic therapy	[103]
Bisphosphonates	Alendronate, Zoledronic Acid	Reduce Mg absorption	Long term use cause Hypomagnesemia and renal impairment	[104]

**Table 3:** Outlines the main non pharmacological causes, mechanism, and related conditions for Mg deficiency.

Etiology	Mechanism	Associated conditions	References
Prolonged Alcohol Use	Reduce Mg intake due to malnutrition, increased gastrointestinal loss and magnesuria	Liver dysfunction, worse liver disease prognosis	[105-107]
Type 2 Diabetes Mellitus	Insulin resistance lead to decreased renal Mg reabsorption and increased magnesuria due to TRPM6 dysfunction	Insulin resistance, hypermagnesuria	[108-110]
Chronic Stress	Mg depletion	-	[111]
Cardiovascular Disease	Abnormal Mg homeostasis increase electrical irritability and risk of arrhythmias with endothelial damage, hemodynamic stress, increase vascular tone, and fibrosis	Elevated BP, hyperaldosteronism	[112, 113]

## LIST OF ABBREVIATIONS

Mg: Magnesium
NMDA: N-methyl-Daspartic Acid
MRS2: Mitochondrial RNA Splicing Protein 2
TAL: Thick Ascending Limb
CaSR: Calcium-Sensing Receptor
AVP: Arginine Vasopressin
DCT: Distal Convoluted Tubule
